# Biochemical and structural characterisation of a protozoan beta-carbonic anhydrase from *Trichomonas vaginalis*

**DOI:** 10.1080/14756366.2020.1774572

**Published:** 2020-06-09

**Authors:** Linda J. Urbański, Anna Di Fiore, Latifeh Azizi, Vesa P. Hytönen, Marianne Kuuslahti, Martina Buonanno, Simona M. Monti, Andrea Angeli, Reza Zolfaghari Emameh, Claudiu T. Supuran, Giuseppina De Simone, Seppo Parkkila

**Affiliations:** a Faculty of Medicine and Health Technology, Tampere University, Tampere, Finland; b Institute of Biostructures and Bioimaging of the National Research Council, Naples, Italy; c Fimlab Ltd, Tampere, Finland; d Neurofarba Department, Sezione di Chimica Farmaceutica e Nutraceutica, Università degli Studi di Firenze, Sesto Fiorentino, Italy; e Department of Energy and Environmental Biotechnology, National Institute of Genetic Engineering and Biotechnology, (NIGEB), Tehran, Iran

**Keywords:** Beta carbonic anhydrase, *Trichomonas vaginalis*, protozoan, kinetics, crystal structure

## Abstract

We report the biochemical and structural characterisation of a beta-carbonic anhydrase (β-CA) from *Trichomonas vaginalis*, a unicellular parasite responsible for one of the world’s leading sexually transmitted infections, trichomoniasis. CAs are ubiquitous metalloenzymes belonging to eight evolutionarily divergent groups (α, β, γ, δ, ζ, η, θ, and ι); humans express only α-CAs, whereas many clinically significant pathogens express only β- and/or γ-CAs. For this reason, the latter two groups of CAs are promising biomedical targets for novel antiinfective agents. The β-CA from *T. vaginalis* (TvaCA1) was recombinantly produced and biochemically characterised. The crystal structure was determined, revealing the canonical dimeric fold of β-CAs and the main features of the enzyme active site. The comparison with the active site of human CA enzymes revealed significant differences that can be exploited for the design of inhibitors selective for the protozoan enzyme with respect to the human ones.

## Introduction

The widespread use of antiinfectives has had a profound impact on global health, causing the development of antiinfective-resistant pathogens. For this reason, new drugs with different mechanisms of action are necessary.

*Trichomonas vaginalis* is a flagellated protozoan parasite that resides in the urogenital tract of men and women^1^. It causes trichomoniasis, one of the most common sexually transmitted infections in the world^2^. Based on a World Health Organisation investigation in 2016, it can be stated that 156 million new trichomoniasis cases emerge every year^3^^,^^4^. This incidence accounts for almost half of the total sexually transmitted infection acquisitions. In women, trichomoniasis usually affects the vagina, but it can also spread to the urethra^5^. The infection typically causes a variety of mild to severe symptoms^1^, with 10–50% of women showing no symptoms^3^ and 5–15% of cases remaining undetectable upon examination^6^. The majority of men infected with *T. vaginalis* are asymptomatic^3^. Mild or nonexistent symptoms make the detection of trichomoniasis particularly challenging, and the infection may go totally unnoticed. The increasing interest in this infection is related to data reporting the relationship between trichomoniasis and other critical pathologies. In fact, it has been shown that infected subjects could exhibit increased susceptibility to human immunodeficiency virus (HIV) acquisition and/or transmission^7^, while in pregnant women, trichomoniasis could induce several complications, such as preterm delivery or premature membrane rupture^8^. Previous studies have suggested that there is an association between *T. vaginalis* and the risk of cervical neoplasia^9^. In addition, new remarkable results have recently been reported on a protein encoded by this infective parasite that is able to drive inflammation and cell proliferation, thus activating molecular pathways that are involved in the promotion and progression of prostate cancer^10^. As a consequence, the diagnosis and effective treatment of *T. vaginalis* infection have become an extremely important goal for global health in both women and men.

A single dose of metronidazole (MET) has been the main treatment against trichomoniasis for nearly five decades^1^. However, the treatment quite often involves multiple rounds of MET^1^, which can lead to a lack of drug compliance and increased antibiotic resistance as the organism adapts and becomes refractory towards the medication. The first reported MET-resistant *T. vaginalis* dates back to 1981^11^. Since then, rare reported cases of MET-resistant strains have emerged^12^. However, the clinical resistance of *T. vaginalis* will likely increase in the future, thus posing a real threat unless novel therapies are discovered.

Alternative approaches for the treatment of trichomoniasis can be developed through the identification of new molecular targets. Among these, carbonic anhydrases (CAs), ubiquitous metalloenzymes present in organisms from all kingdoms of life^13^^,^^14^, have recently emerged. CAs catalyse reversible CO_2_ hydration to bicarbonate and proton. This simple reaction plays an essential role in several physiological processes of microorganism life, such as photosynthesis, CO_2_ transport, pH regulation, and biosynthetic reactions^13^. CAs are divided into eight genetic families: α, β, γ, δ, ζ, η, θ, and ι^14–17^, which can vary in terms of amino acid sequence, oligomeric state, kinetics, and inhibition and activation profiles^13^. α-CAs are the only isoforms present in humans, whereas many pathogens have been discovered with only *β-* and/or *γ-CA* genes in their genome. Based on this observation, these enzymes have been introduced as potential and novel antiinfective drug targets. Indeed, effective inhibitors targeting the active site and thus hindering CA function have been discovered through the production and characterisation of pathogen-specific β- and/or γ-CAs^18–24^.

The analysis of the *T. vaginalis* genome revealed the presence of two *β-CA* genes (TVAG_005270 and TVAG_268150), which encode two proteins, TvaCA1 and TvaCA2, respectively, that share a very high amino acid sequence identity (approximately 72%)^25^. With the aim of identifying new targets for the development of innovative drugs against trichomoniasis, we started our studies on TvaCA1. In this paper, we report the cloning, expression, kinetic and structural characterisation of this enzyme. Our results indicate that TvaCA1 represents a novel potential target for antimicrobial therapy against trichomoniasis.

## Materials and methods

### Protein expression

**Figure 1.**
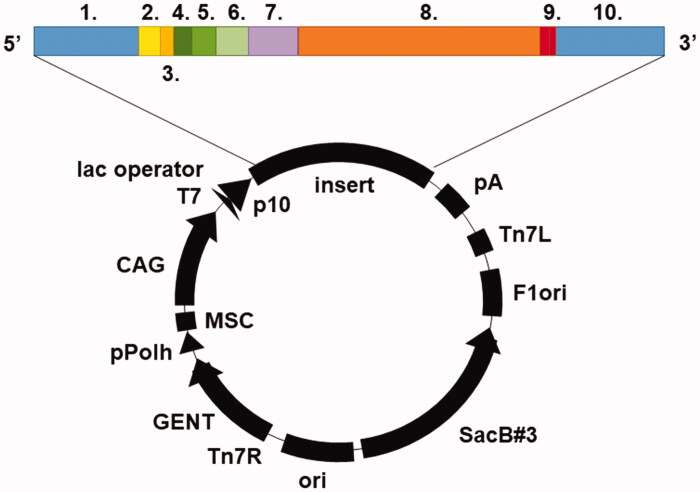
Illustration of the pBVboostFG expression vector. The designed parts of the insert: 1. attL1, 2. Shine-Dalgarno, 3. Kozak, 4. Met-Ser-Tyr-Tyr, 5. 6 × His, 6. Asp-Tyr-Asp-Ile-Pro-Thr-Thr, 7. Lys-Val, 8. CA gene of interest, 9. 2 × stop codon, 10. attL2.

The *TvaCA1* gene sequence was retrieved from Universal Protein Resource Database UniProt (protein entry: A2ENQ8). The destination vector was pBVboostFG^26^, and the subcloned insert was composed of Gateway-compatible recombination sites (attL1, attL2), Shine-Dalgarno and Kozak sequences, a 6xHis-tag with surrounding spacer regions (MSTT and ATAIPTT^27^), *TvaCA1*, and a thrombin cleavage site (LVPRGS^28^) (Figure 1). Gene synthesis and subcloning were performed by GeneArt (Thermo Fisher Scientific, Germany). TvaCA1 was expressed recombinantly in *E. coli* (OneShot^®^ BL21 Star™ (DE3) Chemically Competent Cells, #C601003, Thermo Fisher Scientific, Finland). Transformation was performed according to the Thermo Fisher Scientific OneShot^®^ BL21(DE3) Competent Cells manual (part no. 28–0182). Cells were cultured in Luria-Bertani (LB) medium supplemented with 10 mg/mL gentamicin (1:1000, v/v) at 37 °C until an optical density (OD_595_) of 0.4–0.6 was reached. Expression of the protein was induced by adding 1 M isopropyl β-D-1-thiogalactopyranoside (IPTG) 1:1000 (v/v), after which the culturing was continued overnight at 37 °C. The cells were harvested by centrifugation at 5000 × g for 15 min at 4 °C.

### Protein purification

Harvested cells were mechanically disrupted in 50 mM Na_2_HPO_4_, 0.5 M NaCl and 50 mM imidazole buffer pH 8.0 (binding buffer (BB)) with an EmulsiFlex-C3 homogeniser (AVESTIN, Canada). The lysate was centrifuged at 13000 × g for 20 min at 4 °C. The supernatant was diluted with Ni^2+^-NTA agarose affinity chromatography resin (Macherey-Nagel GmbH Co., Germany) and BB (1:≥3 (vol/vol)). The suspension was incubated for 2 h at RT with gentle agitation, followed by overnight incubation at 4 °C without agitation. Subsequently, the resin was washed generously with BB and packed into a chromatography column with an EMD Millipore™ vacuum filtering flask (#XX1004705, Merck, Finland) and filter paper. The protein was eluted from the resin with 50 mM Na_2_HPO_4_, 0.5 M NaCl and 350 mM imidazole (pH 7.0). The 6xHis-tag was removed by thrombin (#RECOMT, Sigma-Aldrich, Finland) according to the Thrombin CleanCleave™ Kit manual (Sigma-Aldrich, Finland), and the tag was separated from the core protein by Ni^2+^-NTA affinity chromatography. The yield of the protein was determined by a NanoDrop One (Thermo Fisher Scientific, Finland). The quality of the purified protein was analysed by SDS-PAGE using a 12% (w/v) polyacrylamide gel and visualised with PageBlue Protein staining solution (Thermo Fisher Scientific, #24620, Finland). The obtained polypeptide bands of the SDS-PAGE gel were excised and identified using tandem mass spectrometry (Meilahti Clinical Proteomics Core Facility, University of Helsinki, Finland). The sample for crystallisation trials was further purified on a Phenomenex Biosep SEC-S2000 300 × 7.8 mm column in the following running buffer: 50 mM Tris-HCl, 150 mM NaCl, 1.0 mM DTT, pH 8.0. Pooled fractions were concentrated on a 5000 MWCO polyethersulfone membrane (Vivaspin 2, Vivascience Sartorius group, VS0211). The quality of purified protein was analysed by 15% SDS-PAGE, and the protein was detected by blue staining solution (Coomassie Brilliant Blue R-250 #1610400).

### Light scattering

Light scattering methods used to determine the M_w_ of TvaCA1 included SLS and DLS combined with SEC. All measurements were performed after His-tag removal. The instrumentation, which simultaneously measured both LS data, consisted of a Malvern Zetasizer (microV) (Malvern Instruments Ltd., Worcestershire, UK) and a liquid chromatography instrument (CBM-20A, Shimadzu Corporation, Kyoto, Japan) equipped with an autosampler (SIL-20A) and UV–VIS (SPD-20A) and fluorescence detectors (RF-20Axs). UV absorption intensity at 280 nm was used for the determination of the protein concentration. Acquired data were processed with Lab Solution Version 5.51 (Shimadzu Corporation) and OmniSec 4.7 (Malvern Instruments Ltd., Worcestershire, UK) software. Two samples of TvaCA1 (total of 0.4 mg, in PBS) were injected into a Superdex 200 5/150 column (GE Healthcare, Uppsala, Sweden) equilibrated with 50 mM NaH_2_PO_4_ and 500 mM NaCl (pH 8) buffer. Measurements were performed within a thermostable chamber at 20 °C, with a flow rate of 0.1 ml/min. The molecular weight of TvaCA1 was determined in two independent ways: first, based on elution time by using a standard curve calculated according to the elution profiles of standard proteins (SEC analysis: alcohol dehydrogenase 150 kDa, b-amylase 200 kDa, bovine serum albumin 66 kDa and CA 29 kDa (Sigma-Aldrich, Inc., St. Louis, MO, USA)), and second, by calibrating the light-scattering detector based on the monomeric peak of BSA and using the light-scattering intensity (SLS) to determine the protein size. The protein concentration was determined with A_280_.

### Kinetics

An Applied Photophysics stopped-flow instrument was used for assaying CA-catalyzed CO_2_ hydration activity. Phenol red (at a concentration of 0.2 mM) was used as a pH indicator, working at the absorbance maximum of 557 nm, with 20 mM Hepes (pH 7.5) as buffer and 20 mM Na_2_SO_4_ (for maintaining constant ionic strength), following the initial rates of the CA-catalyzed CO_2_ hydration reaction for a period of 10 − 100 s. The CO_2_ concentrations ranged from 1.7 to 17 mM for the determination of the kinetic parameters and AAZ inhibition constant. Six traces of the initial 5 − 10% of the reaction were used to determine the initial velocity. The uncatalyzed rates were determined in the same manner and subtracted from the total observed rates. A stock solution of the inhibitor (0.1 mM) was prepared in distilled − deionized water, and dilutions up to 0.01 nM were prepared thereafter with distilled − deionized water. Inhibitor (I) and enzyme (E) solutions were preincubated together for 15 min at room temperature prior to the assay to allow formation of the E − I complex. The inhibition constant was obtained by nonlinear least squares methods using PRISM 3 and represents the means from at least three different determinations.

### Crystallographic studies

TvaCA1 crystals were obtained at 20 °C by the hanging drop vapour diffusion method. The search for initial crystallisation conditions was performed using Crystal Screen, Crystal Screen 2 and Index from Hampton Research^29^. The wells contained 500 μL of precipitant solution, and the drops were prepared by mixing 1 μL of enzyme solution (11 mg/ml) in 50 mM Tris-HCl, pH 8.0, with 1 μL of the reservoir solution. Good conditions for crystallisation were achieved using a precipitant buffer consisting of 30% (w/v) PEG 4000, 0.2 M sodium acetate, 0.1 M Tris-HCl, pH 8.5. Crystals appeared in the drops within 48 h and grew in approximately one week to maximum dimensions of 0.2 × 0.2 × 0.15 mm^3^. Complete X-ray diffraction data were collected at 100 K with a copper rotating anode generator developed by Rigaku and a Rigaku Saturn CCD detector. Prior to cryogenic freezing, crystals were transferred to the precipitant solution with the addition of 15% (v/v) glycerol. Diffraction data were processed and scaled using the programme HKL2000 (HKL Research)^30^. Crystals belonged to the space group P2_1_2_1_2_1_ with unit cell dimensions of a = 47.3 Å, b = 77.3 Å and c = 90.7 Å. The Matthews coefficient (V_M_ = 2.08 Å^3^/Da) indicated that the asymmetric unit contained two molecules, with a solvent content of 41%. Data collection statistics are reported in Table 1.

**Table 1.** Data collection and refinement statistics

Cell parameters	
Space group	P2_1_2_1_2_1_
Cell dimensions (Å)	a = 47.3
	b = 77.3
	c = 90.7
Number of independent molecules	2
Data collection statistics	
Wavelength (Å)	1.54178
Resolution limits (Å)	41.9–2.48
Total reflections	167801
Unique reflections	12403
Redundancy	13.5
Completeness (%)	99.9 (97.9)
R-merge^a^	0.152 (0.572)
Rmeas^b^	0.158 (0.646)
Rpim^c^	0.042 (0.291)
<I>/<*σ*(I)>	15.9 (2.3)
Refinement statistics	
Resolution limits (Å)	41.9–2.48
Rwork^d^ (%)	19.8
Rfree^d^ (%)	25.7
r.m.s.d. from ideal geometry:	
Bond lengths (Å)	0.004
Bond angles (°)	1.0
Number of protein atoms	2790
Number of water molecules	64
Average B factor (Å^2^)	
All atoms	24.14
Protein atoms	25.28
Waters	17.80
PDB accession code	6Y04

^a^ R-merge = Σ_hkl_Σ_i_|I_i_(hkl) – <I(hkl)>|/Σ_hkl_Σ_i_I_i_(hkl), where I_i_(hkl) is the intensity of an observation and < I(hkl)> is the mean value for its unique reflection; summations are over all reflections.

^b^ Rmeas = Σ_hkl_{N(hkl)/[N(hkl)-1]}^1/2^xΣ_i_|I_i_(hkl) – <I(hkl)>|/Σ_hkl_Σ_i_I_i_(hkl).

^c^ Rpim= Σ_hkl_{1/[N(hkl)-1]}^1/2^xΣ_i_|I_i_(hkl) – <I(hkl)>|/Σ_hkl_Σ_i_I_i_(hkl).

^d^ Rwork = Σ_hkl_ǁFo(hkl)| − |Fc(hkl)ǁ/Σ_hkl_|Fo(hkl)| calculated for the working set of reflections. Rfree is calculated as for Rwork, but from data of the test set that was not used for refinement (Test Set Size (%) = 8.0). Values in parentheses are referred to the highest resolution shell (2.52–2.48 Å).

The structure of TvaCA1 was solved by the molecular replacement technique using the programme AMoRe^31^ and the crystallographic structure of the β-CA from the archaeon *M. thermoautotrophicum* (PDB code 1G5C) as a model template^32^. Refinement of the structure was initially performed with the CNS programme^33^^,^^34^, and model building was performed using O^35^. However, since the electronic density maps were poorly defined both in the enzyme N-terminal region and in the loop encompassing the residues 95–104 of chain B, Auto-Rickshaw was used for rounds of automated model building^36^^,^^37^. This approach allowed the complete reconstruction of the model for both monomers, reducing the Rwork and Rfree values to 0.265 and 0.329, respectively. Many cycles of manual rebuilding and positional and temperature factor refinement were then performed using the REFMAC 5.8 programme^38^ in CCP4i^39^. All refinement cycles were performed with the application of noncrystallographic symmetry restraints. The final model contained 64 solvent molecules and presented crystallographic Rwork and Rfree values (in the 41.9–2.48 Å resolution range) of 0.198 and 0.258, respectively. The refinement statistics are summarised in Table 1. Coordinates and structure factors have been deposited in the Protein Data Bank (accession code 6Y04).

## Results

### Protein production and purification

**Figure 2.**
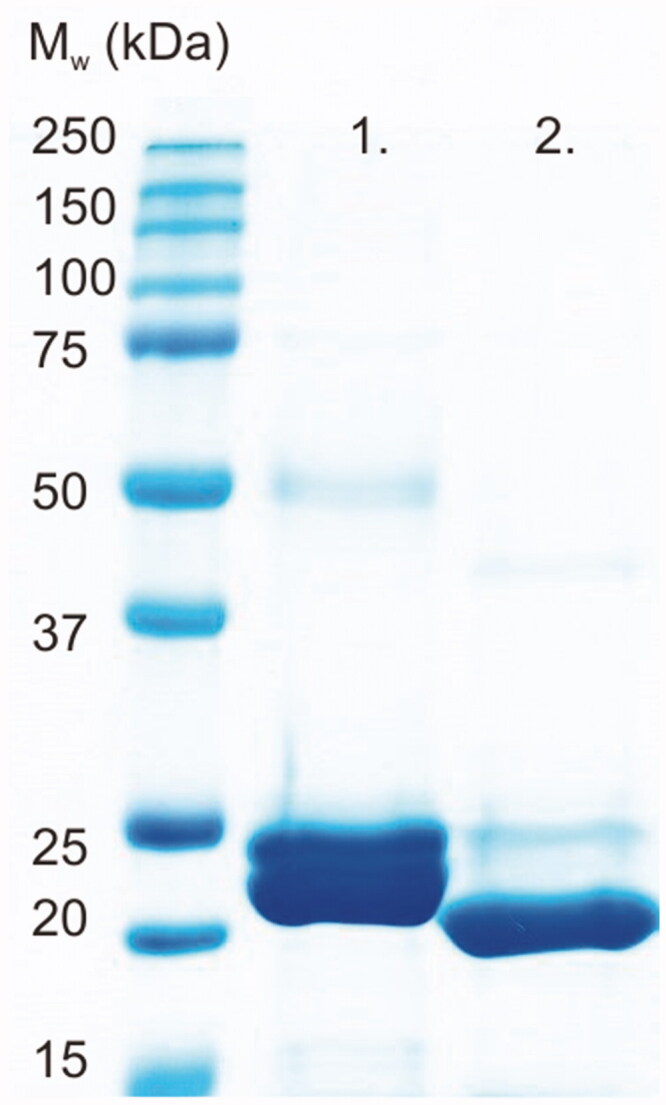
SDS-PAGE of purified TvaCA1 with a 6xHis-tag (lane 1) and after removal of the tag (lane 2). All the polypeptide bands shown on the gel were identified as TvaCA1 protein by MS/MS. The standard molecular weight (M_w_) marker is shown on the far left.

Recombinant TvaCA1 containing a His-tag and a thrombin cleavage site (Figure 1) was expressed in *Escherichia coli* and purified by affinity chromatography, with a yield of approximately 15 mg of purified protein/L of culture. Cleavage of the tag was carried out by thrombin treatment, followed by Protino^®^ nickel-nitrilotriacetic acid (Ni^2+^-NTA) purification, and monitored by sodium dodecyl sulphate polyacrylamide gel electrophoresis (SDS-PAGE) (Figure 2).

### Light scattering

**Figure 3.**
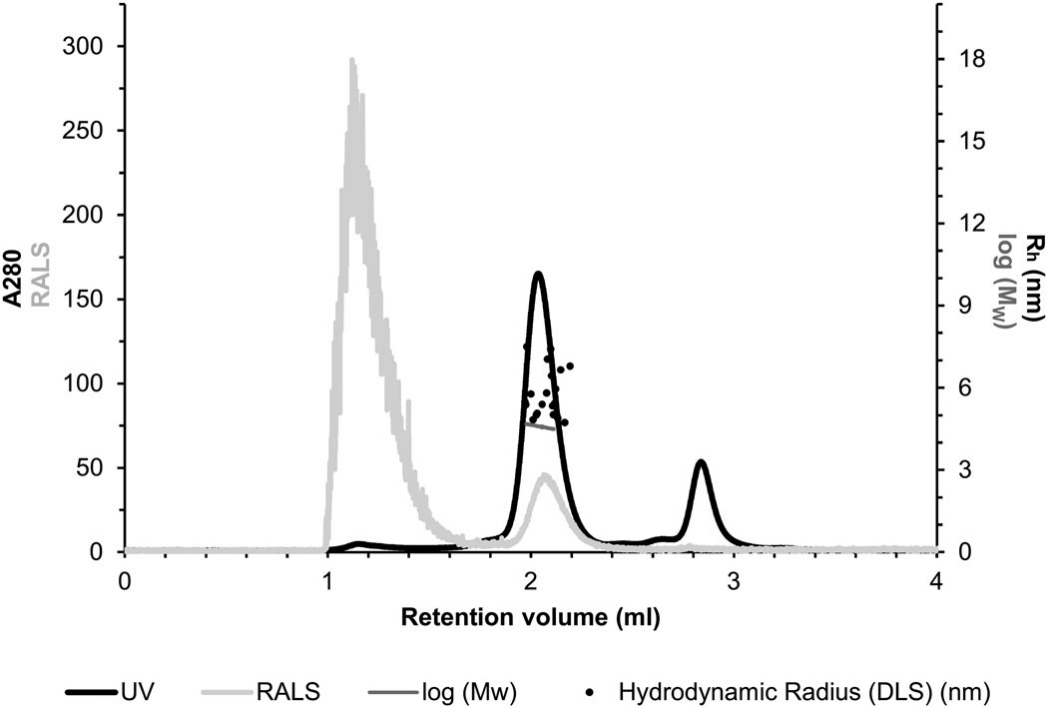
Light scattering data for the assessment of the oligomeric state and size of TvaCA1. The left Y-axis shows the UV absorption intensity at 280 nm and right-angle light scattering intensity (RALS). The right Y-axis shows the M_w_ calculated using static LS intensity.

The quaternary structure of the purified TvaCA1 was investigated by size exclusion chromatography combined with static light scattering/dynamic light scattering (SEC-SLS/DLS). Based on UV absorption at 280 nm (Figure 3, black curve), the main peak was eluted at 2.04 ml. First, the M_w_ of the eluted TvaCA1 was determined using the measured light scattering intensity, and the concentration was determined using UV absorption, resulting in an estimated M_w_ of 39.7 ± 0.4 kDa (Figure 3, horizontal dark grey line across the main peak). Second, the M_w_ was calculated based on elution time by using a M_w_ standard curve and the elution profile of standard proteins, resulting in an estimation of 39.2 ± 0.7 kDa. Both calculations indicated that in our experimental conditions, the native protein is dimeric.

### Kinetics

The kinetic parameters of TvaCA1 are presented in Table 2 and compared with those of human CA isoforms I (hCA I) and II (hCA II).

**Table 2.** Kinetic data of TvaCA1. For comparison, kinetic parameters of hCA I, hCA II, and other representative β-CA enzymes are shown.

Enzyme	k_cat_ (s^–1^)	k_cat_/K_M_ (M^–1^ s^–1^)	*K_i_* (AAZ) (nM)
TvaCA1	4.9 × 10^5^	8.0 × 10^7^	391
hCA I^40^	2.0 × 10^5^	5.0 × 10^7^	250
hCA II^40^	1.4 × 10^6^	1.5 × 10^8^	12
SenCA1^41^	1.0 × 10^6^	8.3 × 10^6^	59
SenCA2^41^	7.9 × 10^5^	5.2 × 10^7^	84
LpnCA1^42^	3.4 × 10^5^	4.7 × 10^7^	76
LpnCA2^42^	8.3 × 10^5^	8.5 × 10^7^	72

AAZ: acetazolamide; SenCA: *Salmonella enterica* β-CA; LpnCA: *Legionella pneumophila* β-CA.

### Structural characterization

The structure of TvaCA1 was investigated by X-ray crystallography. Before crystallisation experiments, an additional purification step was performed. In particular, TvaCA1 was purified by SEC to gain a purity level above 98%. Pooled samples were concentrated to 11 mg/mL and used for crystallisation trials. Crystals were obtained with the hanging-drop vapour diffusion method, using PEG 4000 as the precipitant. The crystals belonged to the space group P2_1_2_1_2_1_, with two molecules per asymmetric unit, and diffracted to a 2.48 Å resolution. The structure was solved by molecular replacement using the β-CA from *Methanobacterium thermoautotrophicum* (MtCab; PDB code 1G5C; 36.3% sequence identity) as the initial model^32^ and refined with the CNS *1.3*^33^^,^^34^ and REFMAC 5.8 programmes^38^ to Rwork and Rfree values of 19.8% and 25.7%, respectively. The refined structure presented a good geometry, with r.m.s.d. from ideal bond lengths and angles of 0.004 Å and 1.0°, respectively. The refinement statistics are summarised in Table 1.

**Figure 4.**
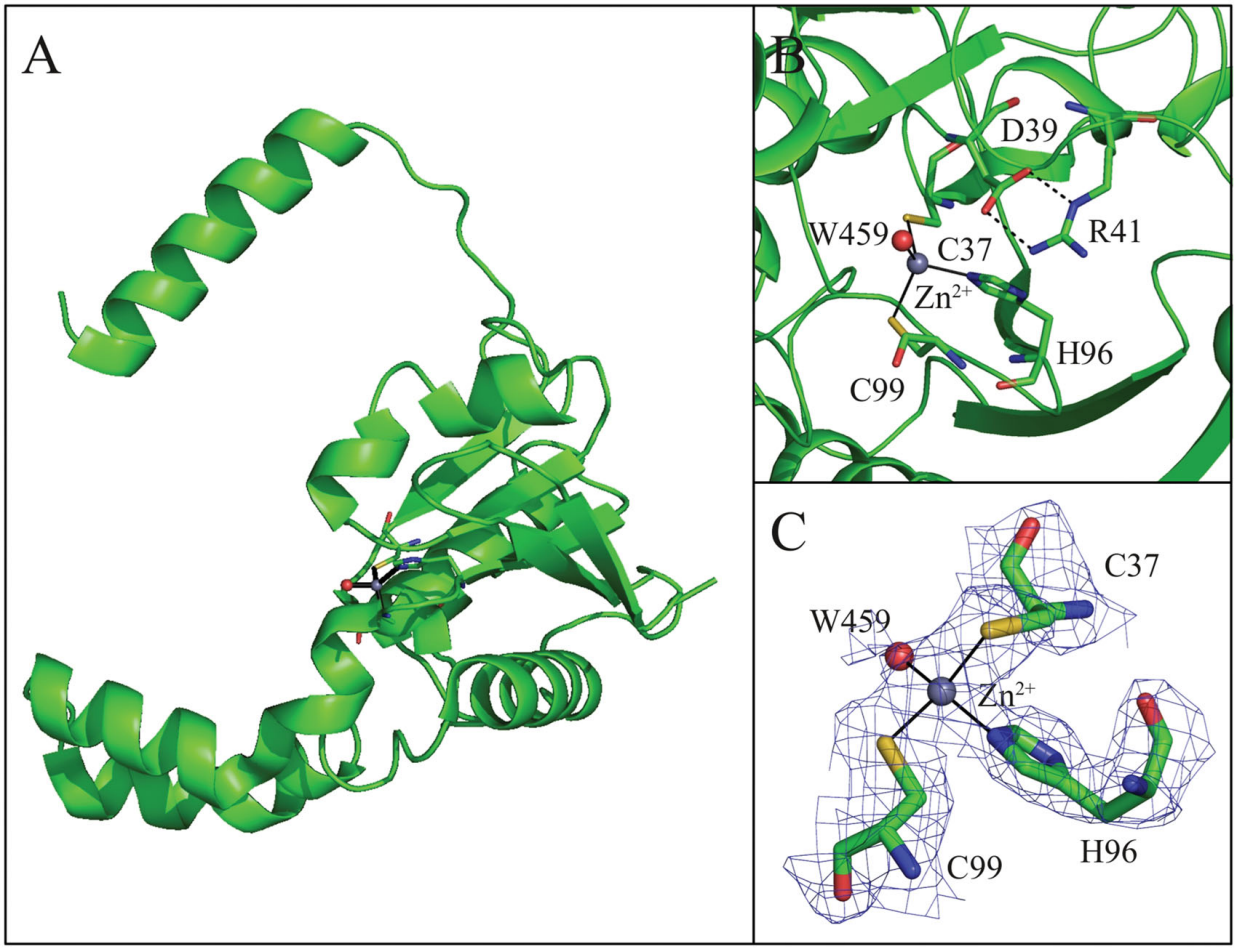
**(**A) Ribbon representation of the TvaCA1 monomer. (B) Enlarged view of the active site, showing Zn^2+^ coordination. (C) σA-weighted |2Fo-Fc| electron density map (contoured at 1.0 σ) relative to zinc ion coordination site.

**Figure 5.**
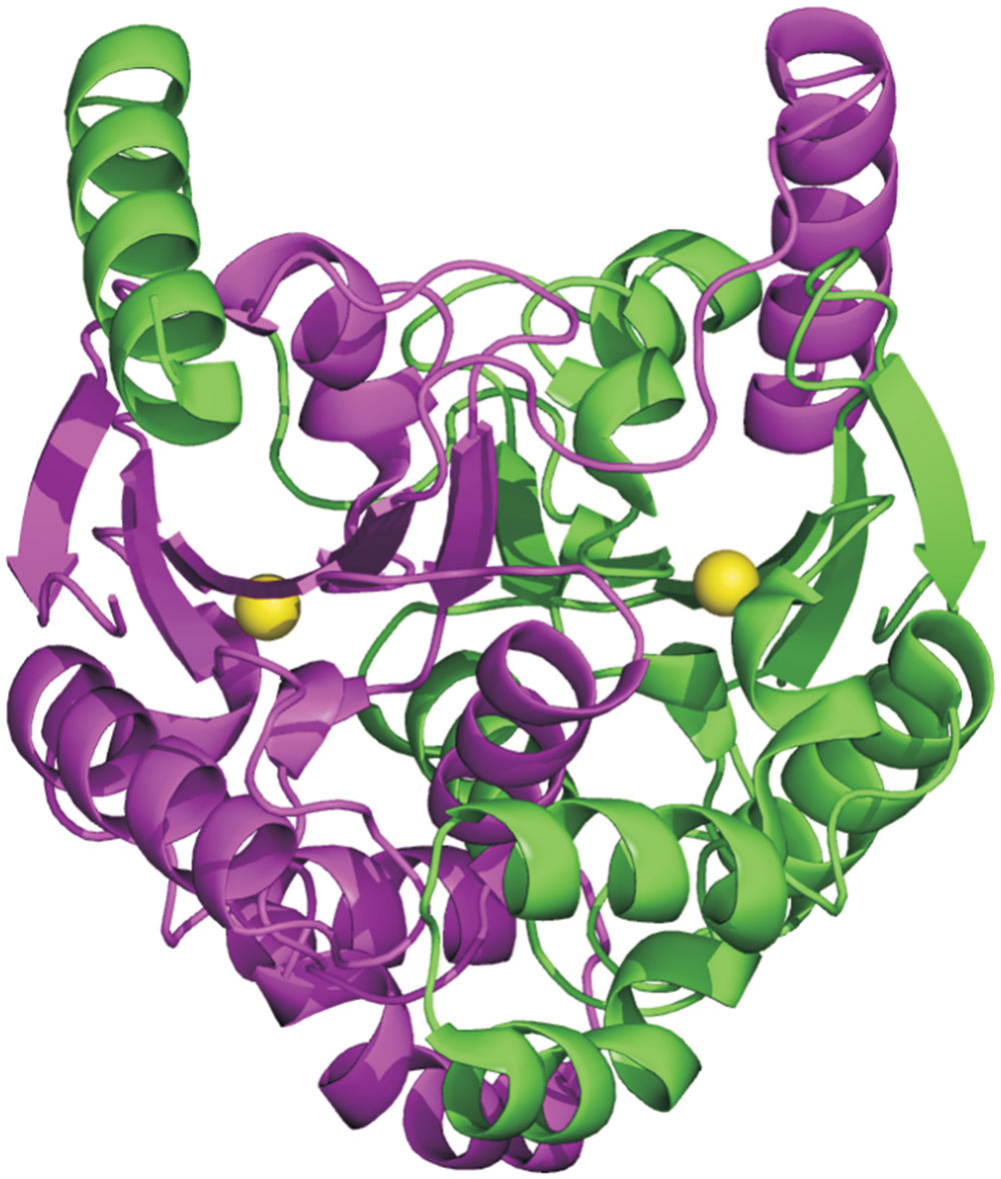
Dimeric structure of TvaCA1, with one monomer coloured in magenta and the other in green. The catalytic zinc ions are depicted as yellow spheres.

TvaCA1 shows the typical α/β-fold observed for other β-CAs^32^^,^^43–54^, consisting of a central mixed five-stranded β-sheet surrounded by several α-helices (Figure 4(A)). In agreement with light-scattering experiment results, the two molecules in the asymmetric unit form a tightly associated dimer characterised by a buried surface area of approximately 4366 Å^2^ (Figure 5) and many hydrogen bonds and van der Waals interactions at the interface between subunits.

The dimer formation produces an extended β-sheet core consisting of ten β–strands, where the N-terminal helix of each monomer extends around the other monomer (Figure 5). There are two active sites per dimer, which are located in clefts at the dimeric interface. Each active site contains a zinc ion on the bottom, which is coordinated by three protein residues, Cys37, His96 and Cys99. In one of the two active sites, a water molecule is clearly visible in the fourth coordination position (Figure 4(B,C)), whereas in the other active site, this water molecule is not visible, probably due to the lower quality of the electron density maps in this region.

Since the first crystallographic structure of a β-CA from the red alga *Porphyridium purpureum* in 2000^48^, 18 other distinct β-CA structures have been reported, belonging to different kingdoms, i.e. plants, fungi, archaeon bacteria and eubacteria (Table 3)^32^^,^^43–55^. Although the sequence alignment of TvaCA1 with all these proteins does not show a very high sequence identity, substantial conservation of the three-dimensional structure is observed, with the highest similarity detected with MtCab^32^, as determined by the DALI server^58^. The main structural differences can be observed in the loops connecting the central β-strands and in the N- and C-terminal regions.

**Table 3.** β-CAs whose crystal structure has been determined

Protein name	Source	Subclass	Assembly	PDB code
PsCA	*Pisum sativum*	Type I	Octamer	1EKJ^43^
CoCA	*Coccomyxa sp.*	Type I	Tetramer	3UCO^47^
ScCA	*Saccharomyces cerevisiae*	Type I	Dimer	3EYX^46^
CAS1	*Sordaria macrospora*	Type I	Tetramer	4O1J^55^
MtCab	*Methanobacterium thermoautotrophicum*	Type I	Dimer	1G5C^32^
Rv1284	*Mycobacterium tuberculosis*	Type I	Dimer	1YLK^44^
HnCA	*Halothiobacillus neapolitanus*	Type I	Dimer	2FGY^45^
CcaA	*Synechocystis sp. PCC 6803*	Type I	Hexamer	5SWC^54^
CafC	*Aspergillus fumigatus*	Type I	Dimer	6JQC^56^
CaNce103p	*Candida albicans*	Type I	Tetramer	6GWU^57^
PpCA	*Porphyridium purpureum*	Type II	Dimer	1DDZ^48^
Can2	*Cryptococcus neoformans*	Type II	Dimer	2W3Q^51^
CAS2	*Sordaria macrospora*	Type II	Tetramer	4O1K^55^
EcCA	*Escherichia coli*	Type II	Tetramer	1I6P^49^
HiCA	*Haemophilus influenzae*	Type II	Tetramer	2A8D^50^
Rv3588c	*Mycobacterium tuberculosis*	Type II	Dimer	1YM3^44^
VchCA	*Vibrio cholerae*	Type II	Tetramer	5CXK^52^
psCA3	*Pseudomonas aeruginosa*	Type II	Dimer	4RXY^53^
TvaCA1	*Trichomonas vaginalis*	Type I	Dimer	6Y04

## Discussion

Compelling data in the literature indicate that interference with CA activity in various protozoan parasites causes impairment of parasite growth and virulence, which in turn leads to a significant antiinfective effect^59–61^. These data, together with the observation that β-CAs are not present in humans, indicate the latter enzymes as excellent targets for the development of new antiparasitic drugs. However, despite their growing importance, only a few papers on the kinetics and inhibition profiles of β-CAs have been published^62–64^, and no crystal structures of a protozoan CA have so far been reported. Here, we illustrate a full biochemical characterisation of TvaCA1, together with its crystallographic structure, thus providing the first detailed characterisation of a protozoan β–CA. In detail, TvaCA1 was expressed in *E. coli*, purified with high yield and kinetically characterised, showing a significant catalytic efficiency comparable to that of known prokaryotic β-CAs, such as *Salmonella enterica*^41^ and *Legionella pneumophila*^42^ (Table 2). Inhibition experiments showed that similarly to CAs from other sources, TvaCA1 is inhibited from the well-known CA inhibitor acetazolamide. Light scattering analysis indicated a dimeric quaternary structure. This finding is in agreement with previous reports on β-CAs, which always show a dimeric structure that in some cases can arrange in higher oligomers, such as tetramers, hexamers or octamers (see Table 3). Accordingly, the crystallographic structure of the enzyme shows the typical dimeric arrangement of β-CAs, characterised by a central β-sheet consisting of 8–10 strands surrounded by several helices.

As observed for other β-CAs so far structurally characterised (Table 3), the TvaCA1 active site is located in a narrow cavity spanning from the protein surface to the catalytic zinc ion. To date, two different subclasses of β-CA enzymes have been identified, which differ in the structural organisation of the active site. Type I β-CAs show a catalytic zinc ion coordination sphere consisting of three protein residues (two Cys and one His) and a water molecule. This kind of coordination was termed “open”, indicating the possibility of performing the catalytic reaction. In type II β-CAs, instead of the water molecule, the metal ion is coordinated to a fourth protein ligand, an Asp residue, resulting in a “closed” metal coordination sphere (Table 3)^65^^,^^66^. Our crystallographic analysis clearly indicates that TvaCA1 belongs to the type I subclass. Indeed, even if the water molecule in the fourth coordination position is clearly visible only in one active site of the dimer (see Figure 4(B)), the aspartic acid residue, which is coordinated to the zinc ion in Type II β-CAs (Asp39 in the TvaCA1 sequence), is in both TvaCA1 active sites, very well defined in the electron density maps and far from the catalytic metal, leaving the active site in the open conformation.

**Figure 6.**
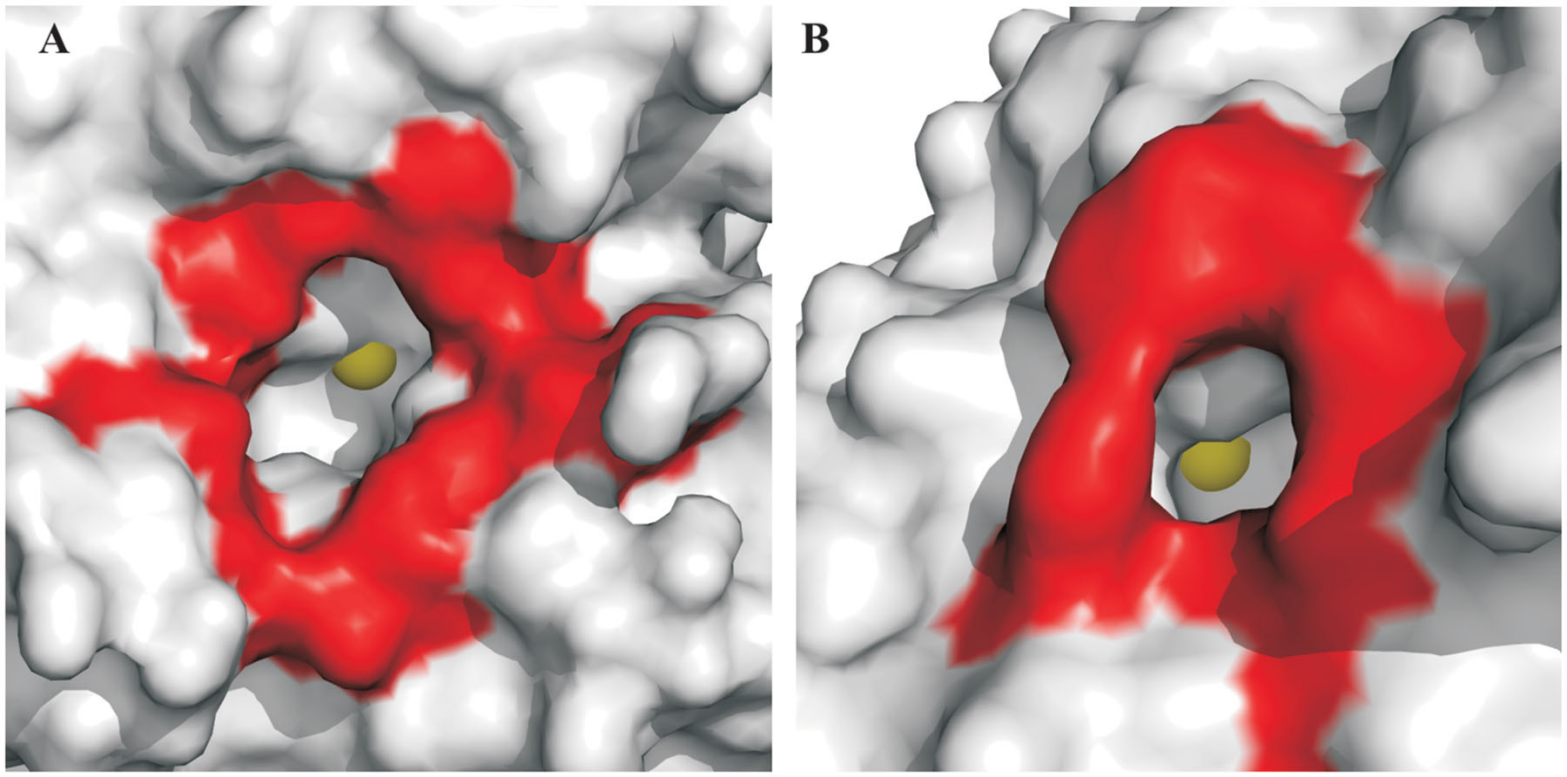
Surface representation of (A) hCA II, chosen as a representative hCA isoform, and (B) TvaCA1. Residues delimiting the rim of the active site cavity are coloured in red. The metal ions are shown as yellow spheres. It is evident that in hCA II, the active site rim is larger (approximately 15 Å × 14 Å) and more accessible than that in TvaCA1 (8 Å × 6.5 Å).

The comparison between the TvaCA1 catalytic cavity and that of human CAs showed significant differences in dimensions (Figure 6), being the latter much larger and more accessible. This finding is particularly important for the development of drugs against trichomoniasis, since these differences can be exploited for the design of inhibitors selective for the protozoan enzyme with respect to the human CAs, which represent an off target. Further studies are currently underway to test this hypothesis.

Taken together, data here reported demonstrate that TvaCA1 is a druggable target and that its selective inhibition is feasible, with the aim of obtaining new antitrichomoniasis drugs.

## Conclusion

In this study, TvaCA1 was successfully expressed in *E. coli,* purified by means of affinity chromatography, tested for kinetic and inhibitory properties, and characterised by X-ray diffraction studies, thus providing the first structural characterisation of a protozoan β-CA. The enzyme was demonstrated to be a noncovalently linked dimer with a narrow cavity leading to the active site. TvaCA1 possessed significant catalytic activity for the CO_2_ hydration reaction and is inhibited by the CA inhibitor acetazolamide. Significant differences between the active site of TvaCA1 and that of human CAs were observed that could be exploited for the design of selective inhibitors for the protozoan enzyme.
